# Survival of cervical cancer patients at Moi teaching and Referral Hospital, Eldoret in western Kenya

**DOI:** 10.1186/s12885-023-11506-w

**Published:** 2023-11-13

**Authors:** E. Mwaliko, P. Itsura, A. Keter, Dirk De Bacquer, N. Buziba, H. Bastiaens, A. Jackie, A. Obala, V. Naanyu, P. Gichangi, M. Temmerman

**Affiliations:** 1 Department of Reproductive Health, School of Medicine, Moi University, Box 4606, Eldoret, 30100 Kenya; 2 Department of Reproductive Health, Gyn-Oncology Group, School of Medicine, Moi University, Box 4606, Eldoret, 30100 Kenya; 3 USAID AMPATH, Moi Teaching and Referral Hospital, Eldoret, Kenya; 4 Department of Public Health and Primary Care, Ghent University, Gent, Belgium; 5 Department of Pathology, School of Medicine/Head, Registry, Moi, Eldoret Cancer , University, Box 4606, Eldoret, 30100 Kenya; 6 Department of Primary and Interdisciplinary Care, Faculty of Medicine and Health Sciences, University of Antwerp, Gouverneur Kinsbergen Centrum, Doornstraat 331, Wilrijk, 2610 Antwerp, Belgium; 7 SBT Population Health AMPATH, P.O. Box 4606, Eldoret, Kenya; 8 Department of Microbiology, Health Sciences Project-VLIR-Moi University Project, P.O. Box 3900, Eldoret, 30100 Kenya; 9 DVC Academic Research & Extension, Technical University of Mombasa/Visiting Professor, Ghent University, Gent, Belgium; 10 Department of Sociology Psychology and Anthropology, School of Arts and Social Sciences, Moi University, P.O. Box 3900, Eldoret, 30100 Kenya; 11 Faculty of Heath Sciences, Department of Obstetrics and Gynaecology Aga Khan University, P O. Box 00100, Nairobi, Kenya

**Keywords:** Cervical cancer, Survival, Kenya

## Abstract

**Background:**

Cervical cancer is a major health burden and the second most common cancer after breast cancer among women in Kenya. Worldwide cervical cancer constitutes 3.1% of all cancer cases. Mortality rates are greatest among the low-income countries because of lack of awareness, screening and early-detection programs and adequate treatment facilities.

The main aim was to estimate survival and determine survival predictors of women with cervical cancer and limited resources in western Kenya.

**Methods:**

Retrospective charts review of women diagnosed with cervical cancer and follow-up for two years from the date of the histologic diagnosis. The outcome of interest was death or survival at two years. Kaplan Meier estimates of survival, log-rank test and Cox proportional hazards regression were used in the survival analysis.

**Results:**

One hundred and sixty-two (162) participants were included in the review. The median duration was 0.8 (interquartile range (IQR) 0.3, 1.6) years. The mean age at diagnosis was 50.6 years (SD12.5). The mean parity was 5.9 (SD 2.6). Fifty percent (50%) did not have health insurance. Twenty six percent (26%) used hormonal contraceptives, 25.9% were HIV positive and 70% of them were on anti-retroviral treatment.

The participants were followed up for 152.6 person years. Of the 162 women in the study, 70 (43.2%) died giving an overall incidence rate (IR) of 45.9 deaths per 100 person years of follow up. The hazard ratios were better for the patients who survived (0.44 vs 0.88, *p*-value < 0.001), those who had medical insurance (0.70 vs 0.48, *p*-value = 0.007) and those with early stage at diagnosis (0.88 vs 0.39, *p*-value < 0.001). Participants who were diagnosed at late stage of the disease according to the International Federation of Gynecology and Obstetrics staging for cervical cancer (FIGO stage 2B-4B) had more than eight times increased hazard of death compared to those who were diagnosed at early stage (1-2A): Hazard Ratio: 8.01 (95% CI 3.65, 17.57). Similarly, those who underwent surgical management had 84% reduced hazard of mortality compared to those who were referred for other modes of care: HR: 0.16 (95% CI: 0.07, 0.38).

**Conclusion:**

Majority of the participants were diagnosed late after presenting with symptoms. The 1 and 2-year survival probability after diagnosis of cervical cancer was 57% AND 45% respectively. It is imperative that women present early since surgery gives better prognosis or better still screening of all women prioritized.

**Supplementary Information:**

The online version contains supplementary material available at 10.1186/s12885-023-11506-w.

## Background

Kenya has a high incidence and mortality from cancer of the cervix. The estimates were 5236 (19.7%) out of all new cases of cancer in women. Cervical cancer was the leading cause of female cancer related mortality. Worldwide there were 9.2 million new cases of cancer in women. Of this, 6.5% were cases of cervical cancer (604,127 new cases). Cervical cancer is the leading cause of cancer deaths followed by cancer of the breast [1]. In contrast to developed countries, there is low rate of survival from cervical cancer in low and low middle income countries (LMICs) with very high rates in sub-Saharan Africa. Various reasons have contributed to this high cervical cancer mortality. There is lack of or low coverage of national screening services. In Kenya only 14% have had screening despite the high cervical cancer awareness rate of 75%. This leads to lack of identification of women at risk or lack of early detection of invasive cancers [2].

More than 90% women in Kenya with cancer of the cervix are diagnosed at advanced stages—according to the International Federation of Gynecology and Obstetrics staging system for cancers (Additional file 1) and usually in health facilities lacking effective treatment. Therefore, there are limited options available for treatment: mainly initial evaluation, symptomatic treatment and referral [3].

In Kenya, at the time of the study there was only one public referral hospital Kenyatta National hospital in the capital city Nairobi that could offer radiotherapy. Access to this hospital is limited by the socioeconomic state of the rural populations. Most patients from the peripheral hospitals are referred to this one hospital that has a backlog of many patients. The number of patients who eventually reach and get radiotherapy can only be speculated. And this is despite the fact that the stage at diagnosis is 2B and above. Three private hospitals at the time of the study were able to offer radiotherapy but at a cost beyond the reach of many patients [4]. The health ministry is in the process of establishing radiotherapy centres at several centres including Moi Teaching and Referral Hospital (MTRH) in western Kenya where this study was undertaken [5].

Late diagnosis is the result of unawareness of cancer and symptom recognition by both patient and health care providers at the primary care level. This, coupled with the time to initiation of radiotherapy leads to worse outcomes as the radiotherapy initiation can take several months [4].

The aim of the study was to determine the survival rate and predictors of survival of cervical cancer patients after diagnosis in western Kenya. More specifically, the study estimated the 1- and 2-year survival and predictors of survival after diagnosis among patients with cervical cancer. An understanding of the contribution of late diagnosis, the lag period to initiation of radiotherapy and their contributions to outcomes has implications on the kind of measures that need to be taken to improve the policy on reducing cervical cancer and its mortality [6, 7].

## Methods

This was a retrospective cohort study with a prospective follow-up data of histologically proven diagnosis of cervical cancer at MTRH in western Kenya. Recruitment was done in the year 2014 and follow-up period was two years.

The study population included all patients diagnosed with cervical cancer that were either admitted in the ward or followed up in the gynecology outpatient clinic or registered in the Eldoret Cancer Registry that is also located within the hospital. The risk factors that were looked for in the charts included: age at first pregnancy, multiple sexual partners smoking, HIV status, post-menopausal state and contraceptive use. The covariates included occupation, education level parity, marital status and health insurance. Human pappiloma status testing was not available.

### Study setting:

The study was conducted at the gynecology-oncology ward and gynecology-oncology clinic/follow up clinic at Moi Teaching and Referral Hospital (MTRH) in Eldoret, Kenya. It is the second largest public teaching and referral hospital in Kenya and the main referral hospital in western Kenya. It has a catchment population of 13 to 15 million people that comprises about 40% of the Kenyan population.

The Eldoret Cancer Registry (ECR) was established in 1999 within MTRH. It collects data on all patients diagnosed with cancer seen at MTRH and thus serves as a hospital-based cancer registry. ECR also collects data from other neighboring facilities with cancer patients, so it is also a population-based cancer registry.

### Inclusion

Women seeking care at MTRH with a histologic diagnosis of cervical cancer were included 1^st^ December 2014. The study concluded on 30^th^ November 2017. Each patient was followed up for two years after recruitment.

### Recruitment of participants

Patients’ charts were retrieved from three sources: first from those identified from the ward registers. Every admitted patient is usually registered and the diagnosis noted. The diagnosis was confirmed by the histology report in the chart. If the biopsy was done during the admission, then the result would be followed up through the outpatient gynecology clinic. Second, if the patient was diagnosed in the outpatient gynecology clinic (usually through screening or came with symptoms) they are usually registered in the computer within the gynecology oncology records department. The records clerk therefore retrieved the files for the needed information. Finally, all cancer cases within the region are recorded in the Eldoret Cancer Registry.

File numbers within the registry are harmonized with the follow-up file numbers in the clinics/ward. The histology results were ascertained to be available and the result recorded. Follow-up information was obtained from files or through phone calls with consent of the patient or relatives for those from other health facilities.

At MTRH, evaluation of patients starts either in the clinic or once admitted in the ward via emergency room. History of symptoms of cervical cancer are taken, then a thorough physical examination is done. Pelvic examination including speculum examination is done.

Staging was done clinically as per International Federation of Gynecology and Obstetrics (FIGO 2009) clinical staging (Additional file 1).

Survival time was calculated as the time (in months or completed years) between the index date and the date of death and or date of loss to follow-up, or the study termination, whichever was earliest.

Age at diagnosis was defined as the age in completed years on the incidence date. Regarding the clinical extent of disease, the FIGO staging was used. Histologic grade was not available in all histology reports as some laboratories omitted this. Whether or not the patient was diagnosed symptomatically or through screening was also noted.

### Ethical consideration

The study was approved by:1) Moi University and Moi Teaching and Referral Hospital Institutional Research and Ethics Committee (FAN: IREC 1071). 2) Ghent University, Commissie voor Medische Ethiek, ONS KENMERK, PA 2011/019. Informed Verbal consent was obtained from the patient (or relative/caretaker) during telephone follow- up.

All records were kept by the research team only and identifiable data was not included in the analysis and discussion.

### Statistical data analysis

Descriptive statistics such as the mean and the corresponding standard deviation (SD) were used to summarize age and parity, and the median and the corresponding interquartile range (IQR) was used to summarize the follow up time.

Frequencies and the corresponding percentages were used to summarize categorical variables such as marital status, occupation, the stage of the cancer, HIV status, use of antiretroviral therapy, and death among others.

Kaplan–Meier survival curves were used to describe the survival distributions. The survival functions for different groups of participants were compared using the Log-rank test.

The hazard ratios and the corresponding 95% confidence interval were computed for each group of participants. The hazard ratios for the different levels of the categorical variables were compared using Cox Proportional Hazards regression model. The ties in time to failure/death were handled using the Breslow test. The hazard ratio (HR) and the corresponding 95% confidence interval were reported.

Data analysis was done using STATA version 13 SE (College Station, Texas 77,845 USA).

## Results

### Demographic characteristics

A total of 162 participants were enrolled in the study. The mean age was 50.6 (SD: 12.5) years with a minimum and a maximum of 17.0 and 80.0 years respectively. Ten percent were aged 35 years or less. Fifty percent had no health insurance.

Two thirds (67.3%) of the participants were married, and 23.5% were homemakers. The sample constituted 7.3% students. Up to 40.2% of the participants had completed primary education. The mean parity was 5.9 (SD: 2.6) with a minimum of zero and a maximum of 13.0.

Twenty six percent (26.5%) used hormonal, and 14.8% used non-hormonal contraceptives. More than half (58.0%) of the participants were post-menopausal. Majority did not use tobacco (79%).

Seventy one percent (71.4%) of those who were HIV infected were on antiretroviral therapy.

Table 1 presents the clinical characteristics of included patients with cancer. The main histological diagnosis was squamous cell carcinoma, observed in 135 (83.3%) patients, and two-thirds (68.5%) of participants were diagnosed at a late cancer stage.

**Table 1:** Clinical characteristics of the cancer patients

Variable	N (%)
Method of diagnosis, n (%)	
Through symptoms	120 (74.1%)
Screening	29 (17.9%)
Not indicated	13 (8.0%)
Diagnosis, n (%)	
Squamous cell carcinoma	135 (83.3%)
Adenocarcinoma	14 (8.6%)
Adeno-squamous	2 (1.2%)
Clinical diagnosis from VE	8 (4.9%)
CIS	2 (1.2%)
Not indicated	1 (0.6%)
Stage of the cancer, n (%)	
Early	51 (31.5%)
Late	111 (68.5%)
Management, n (%)	
Referred for radiotherapy/palliative care/chemotherapy	125 (77.2%)
Surgery	37 (22.8%)

The outcome results (Table 2) show that 45.1% of the participants died, and the median time of follow up was 21 months (IQR: 1.2, 2.0 years) with a minimum of 1 day and a maximum of 2.0 years. Of those who were censored, 3 (1.9%) died after biopsy.

**Table 2:** Patients outcome

Variable	n (%) or Median (IQR)
Survival status, n (%)	
Alive	89 (54.9%)
Dead	70 (43.2%)
Time from diagnosis to death (Years), Median (IQR)	0.8 (0.3, 1.6)
Range (Min. – Max.)	0.03– 2.0

The results also show that the total follow up time was 152.6 person years, and the total number of deaths was 70. This gives an overall incidence rate of death of 45.9 (95% CI: 36.3, 58.0) per 100 person years of follow up.

At one year (Table 3) the survival probability of the participants was 58.0% (95% CI: 48.0%, 65.0%), and at two years 45.0% (95% CI: 36.0%, 54.0%) were still surviving (85% for women in stages 1 to 2A and 43% for women in stages 2B or more). The median (95% CI) survival time is 1.50 years (0.92 to 2.09 years) and the Mean (95% CI) survival time is 1.29 years (1.16 to 1.41 years) (overall survival).

**Table 3:** Survival probabilities at specific time points

Time (Years)	Beginning Total	Deaths	Survival probability (95% CI)
0.5	105	29	0.79 (0.71, 0.85)
1	72	29	0.58 (0.48, 0.65)
1.5	51	9	0.49 (0.40, 0.57)
2.0	28	3	0.45 (0.36, 0.54)

*CI* Confidence Interval

**Fig. 1:**
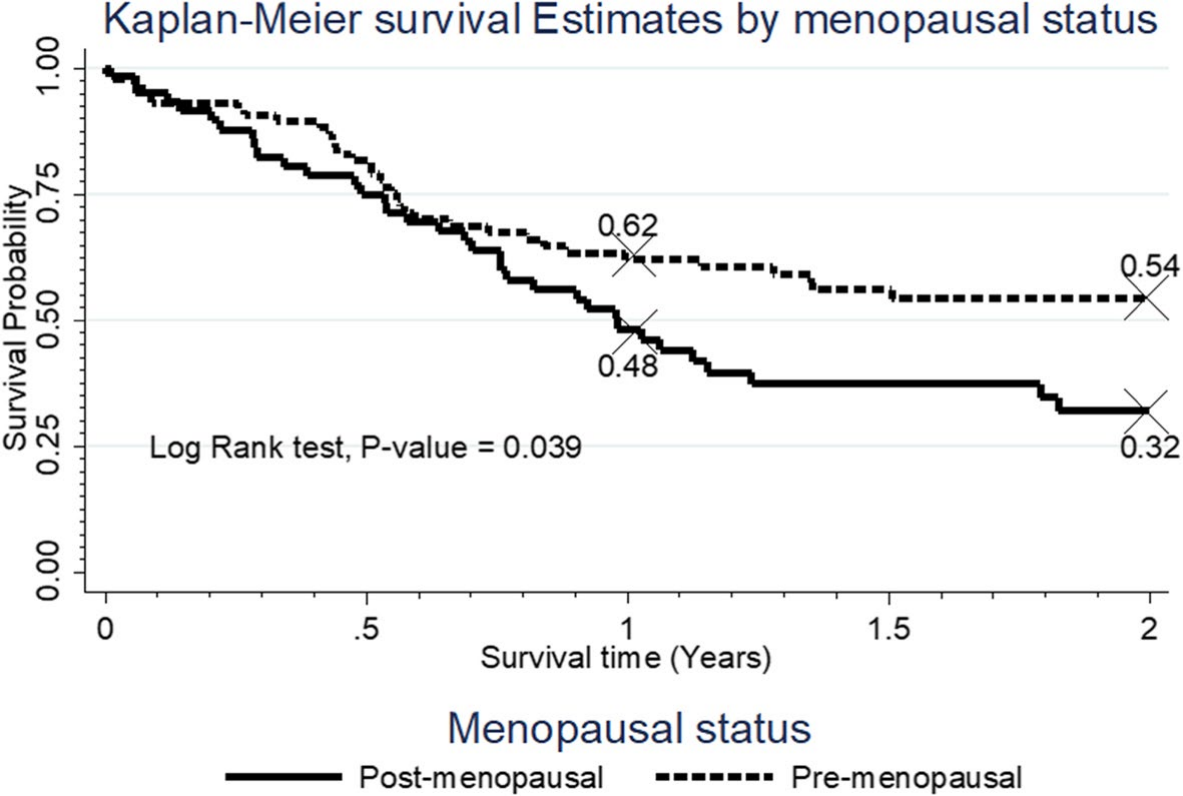
Survival rate by menopausal status

**Fig. 2:**
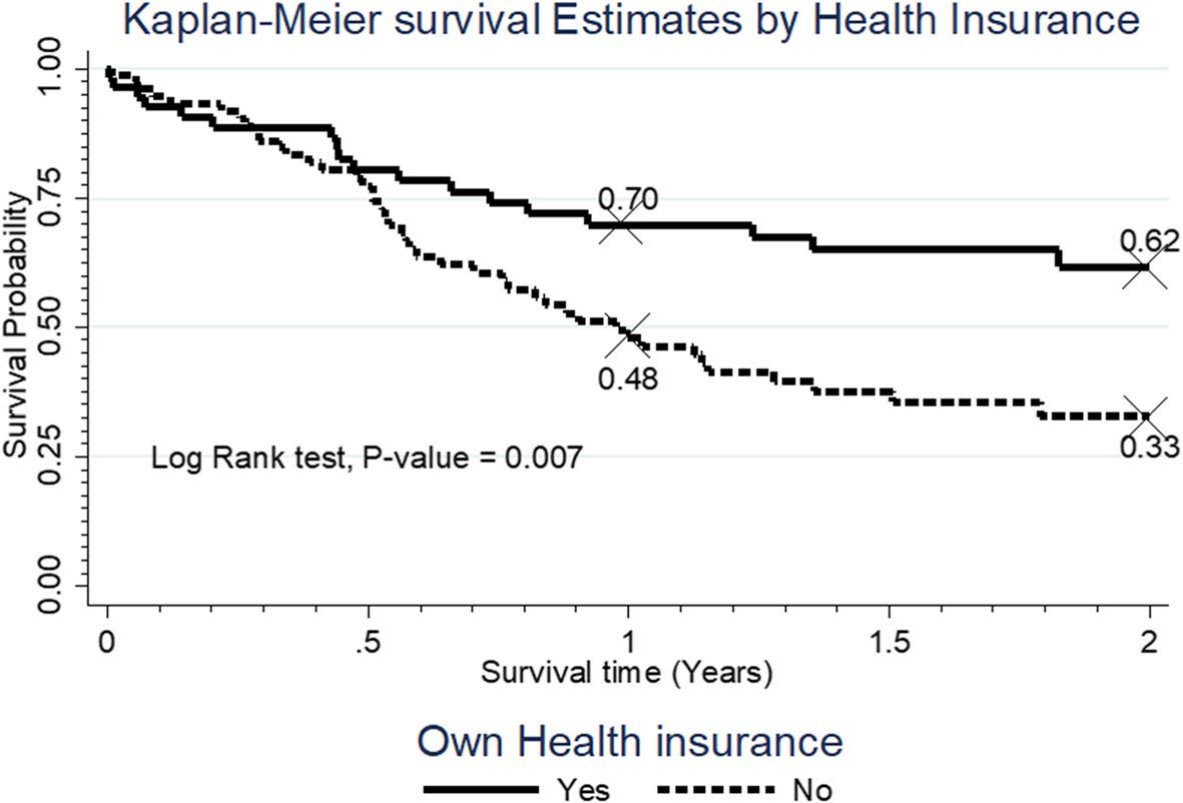
Survival rate by ownership of health insurance

**Fig. 3:**
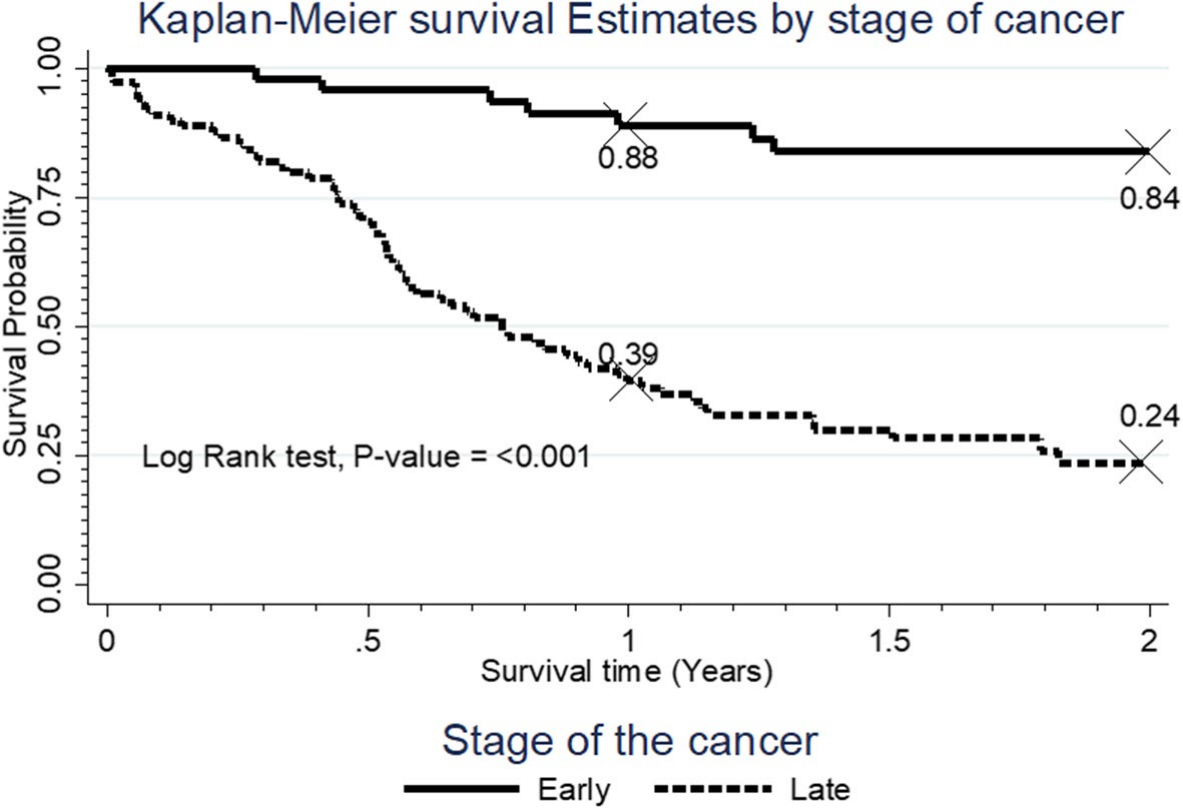
Survival rate by stage of the cancer

**Fig. 4:**
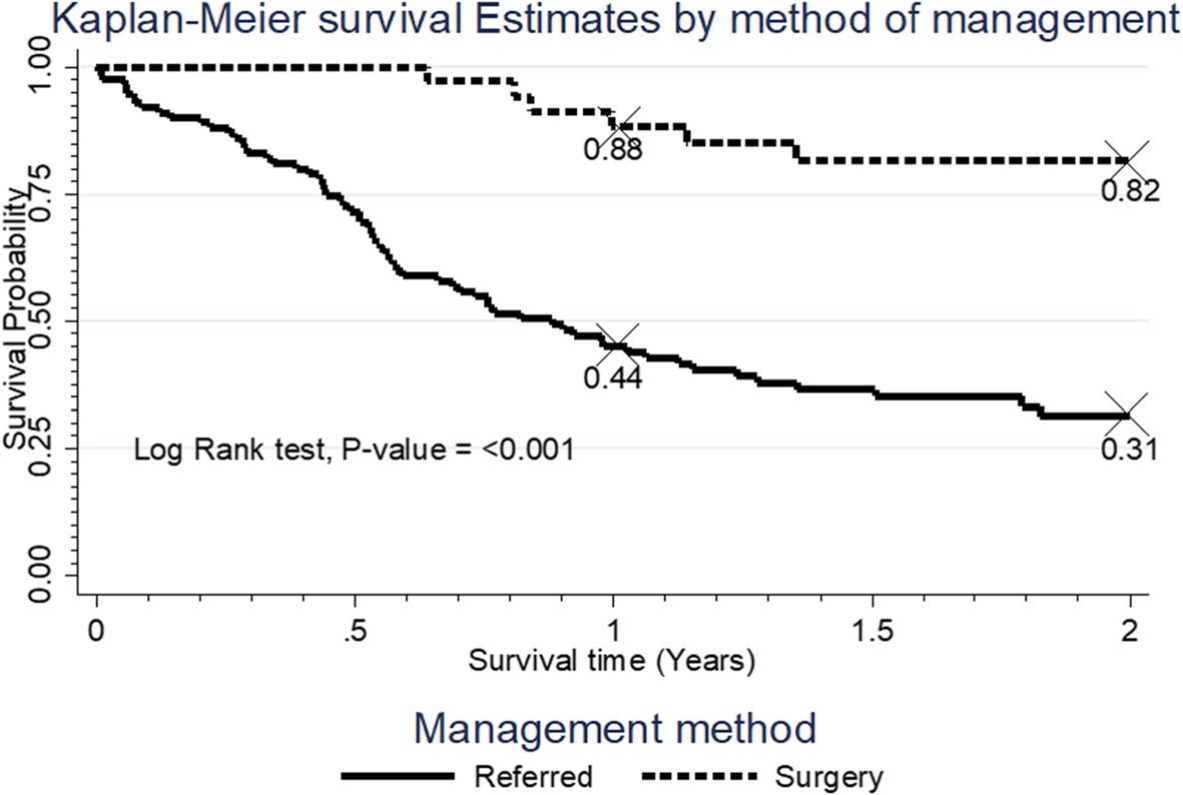
Survival rate by mode of management of the cancer

We explored the survival function of the participants by key variables of interest. The findings are shown in Figs. 1, 2, 3 and 4.

The data shows that the participants who were pre-menopausal had better survival rate compared to those who were post-menopausal, *p* = 0.039 (Fig. 1).

The participants who had health insurance cover had a significantly higher survival distribution compared to those who did not have a health insurance cover, *p* = 0. 007.Up to 70.0% of those who had health insurance cover were still alive at one year compared to 48.0% among those who did not have health insurance cover (Fig. 2).

The participants who diagnosed at an early stage of the cancer had better survival distribution compared to those who were diagnosed late, *p* < 0.001 (Fig. 3).

For women in stages 1 to 2A, the median survival time is undetermined because follow-up was too short to identify a median. The mean (95% CI) survival time was 1.75 years (1.61 to 1.92 years). For women in stages above 2A, the median (95% CI) survival is 0.85 years (0.57 to 1.11 years). The mean (95% CI) survival time was 1.02 years (0.87 to 1.18 years). The probability of surviving 10 months after late-stage diagnosis is 0.5.

The participants who were referred for radiotherapy, palliative care or chemotherapy had a poor survival distribution compared to those who were treated surgically, *p* < 0.001 (Fig. 4).

Table 4 presents the total number of deaths and the corresponding proportion, total follow up time, the incidence rate, and the hazard ratio of death for each level of the categorical variables.

**Table 4:** Incidence and hazard ratio of death

Variable	Deaths (%)	FUP time	Incidence Rate (95% CI) ^a^	Hazard Ratio (95% CI)
Age (Years)				
≤ 35	4 (25.0)	17.6	22.8 (8.5, 60.7)	Reference group
> 35	66 (45.2)	135.0	48.9 (38.4, 62.2)	2.01 (0.73, 5.50)
Education				
Primary/None	31 (47.7)	56.9	54.5 (38.3, 77.5)	Reference group
Secondary/Tertiary	24 (46.2)	54.0	44.4 (29.8, 66.3)	0.87 (0.51, 1.48)
Not indicated	15 (33.3)	41.6	36.0 (21.7, 59.7)	0.70 (0.38, 1.30)
Cohabitation status				
No^b^	20 (48.8)	43.0	46.5 (30.0, 72.2)	Reference group
Yes	47 (43.1)	97.6	48.1 (36.2, 64.1)	0.98 (0.58, 1.65)
Not indicated	3 (25.0)	12.0	25.1 (8.1, 77.8)	0.50 (0.15, 1.70)
Menopausal Status				
Pre-menopausal	35 (37.2)	96.0	36.5 (26.2, 50.8)	Reference group
Post-menopausal	35 (51.5)	56.6	61.9 (44.4, 86.2)	**1.63 (1.02, 2.61)**
Parity				
≤ 5	30 (42.3)	70.7	42.4 (29.7, 60.7)	Reference group
> 5	37 (44.6)	72.2	51.2 (37.1, 70.7)	1.17 (0.73, 1.90)
Have insurance				
No	43 (53.1)	69.0	62.3 (46.2, 84.0)	Reference group
Yes	18 (32.1)	65.4	27.5 (17.4, 43.7)	**0.47 (0.27, 0.82)**
Not indicated	9 (36.0)	18.2	49.4 (25.7, 95.0)	0.78 (0.38, 1.59)
History of contraceptives use				
None	27 (40.3)	59.1	45.7 (31.3, 66.6)	Reference group
Hormonal	15 (34.9)	43.4	34.6 (20.8, 57.4)	0.78 (0.42, 1.47)
Non-hormonal	15 (62.5)	22.8	65.7 (39.6, 109.0)	1.49 (0.79, 2.80)
Not indicated	13 (46.4)	27.3	47.7 (27.7, 82.1)	1.00 (0.51, 1.93)
HIV status				
Not infected	41 (40.6)	95.6	42.9 (31.6, 58.3)	Reference group
Infected	17 (40.5)	41.6	40.8 (25.4, 65.7)	0.95 (0.54, 1.67)
Unknown	12 (63.2)	15.4	78.1 (44.4, 137.6)	1.73 (0.91, 3.30)
Used HAART^c^				
No	1 (33.3)	2.7	36.9 (5.2, 262.3)	Reference group
Yes	13 (43.3)	31.3	41.5 (24.1, 71.5)	1.09 (0.14, 8.32)
Not Indicated	3 (33.3)	7.6	39.3 (12.7, 121.9)	1.13 (0.12, 10.99)
Initial consultation^d^				
Symptoms	54 (45.0)	109.8	49.2 (37.7, 64.2)	Reference group
Screening	8 (27.6)	36.1	22.2 (11.1, 44.3)	**0.47 (0.22, 0.98)**
Not indicated	8 (61.5)	6.6	120.6 (60.3, 241.1)	**2.51 (1.19, 5.27)**
Stage of the cancer				
Early	7 (13.7)	74.3	9.4 (4.5, 19.8)	Reference group
Late	63 (56.8)	78.3	80.5 (62.9, 103.1)	**8.01 (3.65, 17.57)**
Management				
Referred	64 (51.2)	95.4	67.1 (52.5, 85.7)	Reference group
Surgery	6 (16.2)	57.1	10.5 (4.7, 23.4)	**0.16 (0.07, 0.38)**

^a^—Incidence was calculated per 100 person years of follow up, CI – Confidence Interval

^b^Single, Separated, and Widowed

^c^Among the HIV infected; FUP – Follow up time (in years)

^d^-health care seeking reason

The adjusted model (Table 5) shows that the participants who were diagnosed at a late stage of the disease were associated with more than five times increased hazard of death compared to those who diagnosed at an early stage of the disease, HR: 5.20 (95% CI: 2.28, 11.87).

**Table 5:** Unadjusted and adjusted risk factors

Variable	Unadjusted Hazard Ratio (95% CI)	Adjusted Hazard Ratio (95% CI)
Age (Years)		
≤ 35	Reference group	Reference group
> 35	2.01 (0.73, 5.50)	1.12 (0.40, 3.15)
Have insurance		
No	Reference group	Reference group
Yes	**0.47 (0.27, 0.82)**	0.64 (0.37, 1.12)
Not indicated	0.78 (0.38, 1.59)	0.68 (0.32, 1.42)
HIV status		
Not infected	Reference group	Reference group
Infected	0.95 (0.54, 1.67)	1.39 (0.73, 2.66)
Unknown	1.73 (0.91, 3.30)	1.14 (0.64, 2.04)
Stage of the cancer		
Early	Reference group	Reference group
Late	**8.01 (3.65, 17.57)**	**5.20 (2.28, 11.87)**
Management		
Referred	Reference group	Reference group
Surgery	**0.16 (0.07, 0.38)**	**0.36 (0.15, 0.90)**

Similarly, the adjusted effect of management of the disease show that surgical procedure was associated with 64% reduced hazard of mortality compared to those who were referred, HR: 0.36 (95% CI: 0.15, 0.90).

There was no evidence of a difference in the survival rate of the participants between those who had secondary or tertiary and those who had primary level or incomplete primary level of education or those who had no education at all, *p* = 0.526.

There was no difference in the survival distribution of the participants who were HIV infected compared to those who were HIV non-infected, *p* = 0.859. The rate of survival for those who were HIV infected was similar to the rate of survival among those who were HIV non-infected at 1 year and at two years.

## Discussion

This was a retrospective cohort study conducted at a national tertiary hospital. In this study we reviewed 162 patients’ charts. Each patient recruited was to be followed up for a complete two years. In this study 68.5% participants presented with advanced stages which is consistent with other studies in low-income countries [8–11]. The majority of the diagnosis were made following symptomatic presentation. This indicates the lack of screening and the need to take symptoms as important to indicators in earlier diagnosis of cervical cancer. The late presentations also reflect delayed diagnosis due to limited accessibility/availability of oncology services especially in rural areas [12].

This study showed that the 1- and 2-year survival probability was 58% and 45% respectfully. The overall incidence of death of 45.9 per 100 women years. Most of the dead participants died within the first year (80%). The 1-year survival which is a proxy for early diagnosis and also indicative of the stage at diagnosis. The one-year survival for those stage 2A and below was 88%. The probability of surviving 10 months after diagnosis was 0.5 for those in stage 2B and above. Compared to the 5-year survival in developed countries, this still further confirms that most were diagnosed late when effective treatment is not available [13].

The patients were categorized into early (stage 2A and below) and late (above stage 2B). Thirty two percent (32%) of the patients presented in early stage. Late presentation is consistent with other studies done in Africa [8–10]. All these studies highlight the challenge posed by the late presentations and survival in Kenya and Africa as a whole. The one-year survival rates for those diagnosed in early stage was 88% compared to 39% for those diagnosed in late stage.

Survival rate was analyzed by mode of management after diagnosis. Patients either had surgery or were referred for radiotherapy/palliative care. Those who underwent surgery had better survival than those who were referred. Musa and colleagues’ study in Nigeria showed “potential benefit” of surgery [10]. In this study those who were referred had 1- and 2-year survival rates of 44% vs 89% and 31% vs 82% compared to those who underwent surgery. Though the extent of surgery and the complications are controversial the survival benefit it confers in this setup is clinically significant. Health systems in SSA are overwhelmed with many competing priorities and referral option for many patients is unattainable meaning many may not even present themselves for chemo/radiation (lack of finances) and choose traditional medications. Cancer survival after treatment reflects the availability and accessibility of cancer health services in the region.

Ginsberg and colleagues have shown that “treatment approach” costs are higher than “prevention/and early detection and treatment approach”. However, primary prevention using vaccines is not widespread and screening in this region is sporadic at best with low coverage [14].

Therefore, if diagnosed early- which should be the aim, surgery would be sensible in this context.

In the predictive model we also analyzed other factors associated with mortality – age at diagnosis, HIV status and use of HAART, and whether one was diagnosed through symptoms or just incidental during screening.

A Swedish study found that women diagnosed with cervical cancer at age above 65 had more advanced disease compared to those below [15]. This was mainly due to being left out of the screening program and the prognosis was poor. This can be assumed to be the case in our context where no screening program is in place. However, the proportions in our study did not show a significant difference in the hazard ratio. We compared those who were above 35 years to those below 35 years in this study. We had 16 participants under 35. When compared to those above 35 years the incidence rate of death was higher among those above 35 years though it was not significant. This is similar to a study by Pelkofski and colleagues who concluded that age at diagnosis on its own for the under 35 does not infer a worse prognosis [16]. This may be because of the few numbers of participants under 35 that we had in this cohort that did not allow a difference to be noted (there was only one participant aged 17 who was censored alive at 2 years) and we cannot conclude from this study whether cervical cancer is more aggressive in the under 35 years because we got the opposite.

Age at first sexual encounter and age at first full term pregnancy have been shown to be risk factors for cervical cancer [17]. The higher the parity the lower the age at first pregnancy the higher the exposure to HPV therefore the higher the risk. There was no difference however in the incidence rate of death between those who had parity less than 5 compared with those with more than 5 in this study. Age of participants in this study was similar to the study in Ethiopia where the number of those above age 35 was found to be more than the under 35 years [18].

As expected, those diagnosed through screening had reduced mortality rate. Majority would be in early stages at most and therefore more likely to go managed through surgery. And it is the rationale for early detection call for women to undergo screening regularly.

In a study in Malawi which discusses the relationship of HIV with pre invasive lesions and cervical cancer, the incidence of cervical cancer was not found to be different between the infected and non-infected. However, the incidence of precancerous lesions was increased. Survival for HIV infected patients in Botswana was lower than the HIV negative patients [19, 20].

In this study those who were HIV infected had a non-significant lower incidence of death compared with HIV negative participants. We postulate that patients in this referral Centre with facilities for follow-up of all HIV patients including regular screening (integrated services which has been shown to be feasible with reduction of loss to follow-up) of these patients probably enabled early diagnosis in these patients as opposed to HIV negative patients who have to seek for screening and pay for it. When a patient with cervical cancer is HIV positive the prognosis will depend on the stage at diagnosis. Previous studies had shown more toxicity with HIV positive patients undergoing chemo radiotherapy [21, 22]. A study done in Zambia demonstrated no significant difference with regard to major acute reactions between HIV positive on HAART and HIV negative groups [23]. A Brazil study found no association between HIV infection with initial treatment response or early mortality. However, relapse after attaining a complete response and late mortality were increased [24].

In this study there was no evidence of association between use of HAART and reduction in mortality due to cervical cancer among HIV positive patients.

There was no difference from this study that those who used hormonal contraceptive had a different incident rate of death [25]. Survival for those who were premenopausal was better than those who were postmenopausal. This is expected from the data as we had more women elderly and with late stage at diagnosis.

We also analyzed factors associated with loss of follow-up. These were education level, marital status, whether patient have medical insurance or not. These factors are known to increase delay in presentation for diagnosis [26]. Poverty leading to being less educated and lower socioeconomic status leads to low levels of symptom awareness. In this study we examined the hazard ratio of those with and those without health insurance (paid by participant as part of health care in the country). Those who had insurance had lower hazard ratio compared to those who did not. Demands and priorities on finances and also fear of diagnosis have been reported in qualitative studies as reasons for late presentation for diagnosis [27]. Cohabitation status differentials in cervical cancer incidence may reflect differences in socio economic status (SES) especially where women don’t own/inherit from fathers/husbands like in this context, behavioral factors, social networks, and social support characteristics. All these in one way are risk factors for cervical cancer and influence survival –specifically ability of the patient to pay for the cost of treatment after referral to the city.

As has been noted before health systems in SSA are overwhelmed with many competing priorities while poverty in most areas limit the patients’ choice to seek care. Majority do not have health insurance [28, 29].

### Limitations

Though date of diagnosis and recommended treatment was possible the follow-up was difficult. Some of the contact (phone) information given proved to be inaccurate and also some relatives of the patients contacted were reluctant or uncooperative in divulging information.

The size of the sample was small which may have limited the statistical power.

The small cohort size did not allow us to conduct analysis like the effect of HIV/HAART status, surgery vs chemo radiation, hormonal use vs non-hormonal use and different age categories.

## Conclusion

The predictors of death among women diagnosed with cervical cancer in MTRH were stage at diagnosis, mode of management and having health insurance. The overall incidence of death was 45.9 per 100 person years of follow up. And the 1- and 2-year survival was 57% and 45% respectively.

The poor survival can be attributed due to lack of screening and early diagnosis leading to late stage at presentation. Also access to radiotherapy services was difficult for patients as there was only one public facility for this in the country. Basic cancer services are still required for diagnostic, surgery radio/chemotherapy and palliative care and appropriate follow up after diagnosis and treatment. This is urgent considering it will be many years before primary prevention and screening finally achieves its goal.

The start date was defined as the date of the diagnosis and the outcome of interest was death. Complete follow-up was achieved when vital status (alive/dead) at the closing date was known for an individual. We employed active follow-up methods. Information on deaths was sourced from patient’s clinical record files and included repeated scrutiny of medical records. In addition, telephone enquiries to patients or relatives/caretakers whose phone numbers were in the patients file were made.

### Censoring

This occurred either at death, the closing date of the study or were lost to follow-up. Loss to follow-up happened when they did not return to the gynecology clinic or could not be contacted and we could not ascertain whether they were still alive after last known status date. Deaths due to other causes than complications of cervical cancer were not captured in this study.

Index date and closing date to follow-up:

The index date is the starting date for calculation of survival, and this was the date of unequivocal diagnosis of cancer by means of histological diagnosis. The inclusion date was between 1st of December 2014 to 30^th^ November 2017. Each patient was followed up for two years i.e., up to the closing date of the study November 2017 or to death (date of death was reported) or until when they were censored as a result of transfer to other facilities, for home care or loss to follow up.

### Supplementary Information

**Additional file 1. **FIGO Staging System for Uterine Cervical Cancer (2018).

## Data Availability

The datasets used and/or analysed during the study are available from the corresponding author on reasonable request.

## Abbreviations

Median survival time: Point at which half the patients have experienced the event under study and half remain free of the event (in this study death)
Overall survival time: The length of time from the date of diagnosis for a disease that patients are still alive
HAART: Highly Active Antiretroviral Therapy
VE: Vaginal Examination
SES: Socio Economic Status
PI: Principal Investigator
SSA: Sub-Saharan Africa
FIGO: International Federation of Gynecology and Obstetrics staging system for cancers
Early-stage cancer of the cervix: Stage 1-IIA
Late stages cancer of the cervix: Stage IIB-IV
HR: Hazard ratio
CI: Confidence interval
