# Supplementary material for: Survival of cervical cancer patients at Moi teaching and Referral Hospital, Eldoret in western Kenya
Source: BMC Cancer. 2023 Nov 13;23:1104. doi: 10.1186/s12885-023-11506-w (PMC10644535; doi:10.1186/s12885-023-11506-w)
Supplement: Supplementary file 1 — Additional file 1. FIGO Staging System for Uterine Cervical Cancer (2018). [file 12885_2023_11506_MOESM1_ESM.docx]

**Supplementary file:**







• 

a

 

 

• IB

b

 IB1

 IB2

 IB3





• 

 

 

• IIB







c

• 

• IIIB

• 

c

 

 

:





• 

• 



|



FIGO COMMITTEE REPORT

        





 

  

      

       

        







 

         



 



 









Box 1





• 

a

 

 

• IB

b

 IB1

 IB2

 IB3





• 

 

 

• IIB







c

• 

• IIIB

• 

c

 

 

:





• 

• 

a



b



c









|



FIGO COMMITTEE REPORT

        





 

  

      

       

        







 

         



 



 









Box 1





• 

a

 

 

• IB

b

 IB1

 IB2

 IB3





• 

 

 

• IIB







c

• 

• IIIB

• 

c

 

 

:





• 

• 

a



b



c







FIGO Staging System for Uterine Cervical Cancer (2018)

| **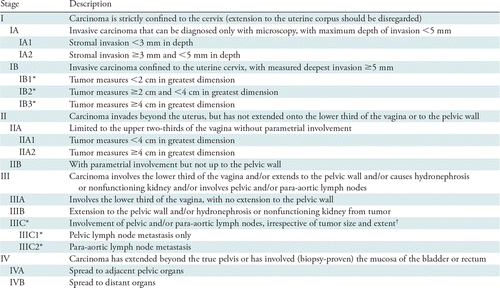** |
| --- |

Note: Imaging and pathologic analysis, where available, can be used to supplement clinical findings for all stages.

FIGO, International Federation of Gynecology and Obstetrics (Adapted, under a CC BY license, from reference 1).

*Indicates stages that are new since the 2009 FIGO system.

^†^ Stage IIIC should be annotated with *r* (radiology) or *p* (pathologic analysis) to indicate the method used to allocate to this stage. Imaging modality or pathologic technique should also be documented.
